# Proteomic analysis and effects on osteogenic differentiation of exosomes from patients with ossification of the spinal ligament

**DOI:** 10.1093/jbmrpl/ziaf021

**Published:** 2025-02-02

**Authors:** Hideaki Nakajima, William E B Johnson, Mikiko Kamitani, Shuji Watanabe, Kazuya Honjoh, Arisa Kubota, Akihiko Matsumine

**Affiliations:** Department of Orthopaedics and Rehabilitation Medicine, Faculty of Medical Sciences, University of Fukui, Yoshida-gun, Fukui 910-1193, Japan; Chester Medical School, University of Chester, Chester CH1 4BJ, United Kingdom; Department of Orthopaedics and Rehabilitation Medicine, Faculty of Medical Sciences, University of Fukui, Yoshida-gun, Fukui 910-1193, Japan; Department of Orthopaedics and Rehabilitation Medicine, Faculty of Medical Sciences, University of Fukui, Yoshida-gun, Fukui 910-1193, Japan; Department of Orthopaedics and Rehabilitation Medicine, Faculty of Medical Sciences, University of Fukui, Yoshida-gun, Fukui 910-1193, Japan; Department of Orthopaedics and Rehabilitation Medicine, Faculty of Medical Sciences, University of Fukui, Yoshida-gun, Fukui 910-1193, Japan; Department of Orthopaedics and Rehabilitation Medicine, Faculty of Medical Sciences, University of Fukui, Yoshida-gun, Fukui 910-1193, Japan

**Keywords:** ossification of the spinal ligament, ossification of the ligamentum flavum, exosome, proteomic analysis, osteogenic differentiation

## Abstract

Ossification of the spinal ligament (OSL), including ossification of the posterior longitudinal ligament and ossification of the ligamentum flavum (OLF), is a multifactorial disease that includes genetic predisposition. The association between the rate of ossification in the spinal canal and the severity of myelopathy symptoms is well known, but the degree of progression varies widely among patients. Although many candidate genes and biomarkers have been reported, there are no definitive and quantitative conclusions to date, probably because of low reproducibility due to individual differences. In this study, we focused on exosomes secreted by ossified spinal ligament cells. Exosomes are crucial for intercellular communication during development and progression of disease. In a co-culture study of non-OLF cells with OLF cells, there was increased osteogenic differentiation, including Runx2 and Wnt3a expression, with use of exosome-penetrating filters (1.2 μm) compared to exosome-non-penetrating filters (0.03 μm). Dose-dependent increases in alkaline phosphatase activity and mineral deposition were observed in non-OLF cells treated with OLF-derived exosomes. These results support the hypothesis that OLF-derived exosomes are involved in regulation of osteogenic differentiation. In comparative proteomics analysis, 32 factors were increased and 40 were decreased in OLF-derived exosomes compared to non-OLF-derived exosomes. Molecular network analysis of these 72 factors indicated 10 significant pathways, including the matrix metalloproteinase (MMP) signaling, mTOR signaling, Wnt signaling and VDR-associated pathways. Among the upregulated exosomal membrane proteins in OLF samples, COL IV, FMNL3, mTORC2, and PIP4K showed increased expression with greater ossification, suggesting they may serve as biomarkers of disease activity and therapeutic targets. These factors are involved in the PI3K/Akt/mTOR signaling pathway, and particularly mTOR is known to regulate osteogenic and chondrogenic differentiation. In contrast, fatty acid-binding protein 5, several KRT family proteins, S100A8, SERPINB3, and transglutaminase, were significantly downregulated in OLF-derived exosomes. These findings provide novel insights into the molecular mechanisms underlying OSL pathogenesis.

## Graphical Abstract

**Graphical Abstract:**
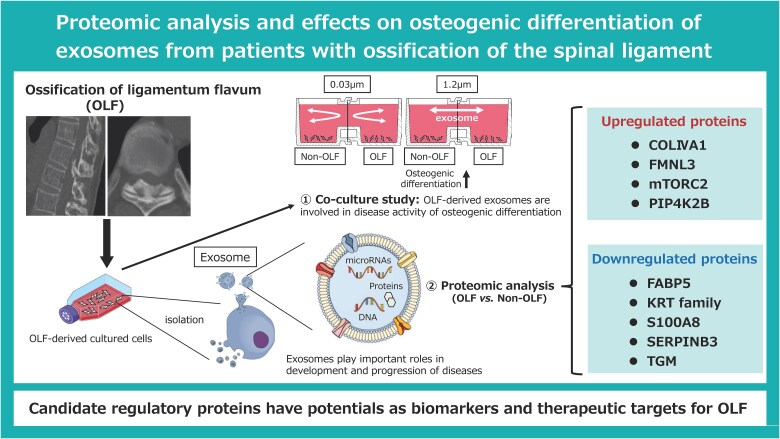

## Introduction

Ossification of the spinal ligament (OSL), including ossification of the posterior longitudinal ligament (OPLL) and ossification of the ligamentum flavum (OLF), refers to a hyperostotic condition of the spine that can lead to gradual loss of function of the spinal cord, including motor dysfunction, sensory disturbance deficits, and neuropathic pain. The prevalence of cervical OPLL in Japan is 6.3%, compared to 0.12% in the U.S. and 0.1% in Germany, and thus, is much higher in Japan and East Asia.^1^ The prevalence of thoracic OLF in Japan is 12%, and cervical OPLL and thoracic OLF can co-exist at a high rate (45%-60%).^2^^,^^3^

An association between the rate of ossification in the spinal canal and the severity of myelopathy symptoms in the patients with OSL has been described, and many studies have examined the etiology and pathogenesis of ossification.^4^^,^^5^ Progression of ossification occurs slowly in the natural course, with the Spinal Ligament Ossification Study Group finding that the ossification lesion increased by 61% in the longitudinal direction and 31% in thickness over a 10-yr follow-up period.^6^ Another study using CT found an average annual volume increase of 4.1%.^7^ For thoracic OLF, cross-sectional studies have shown a higher rate of ossification in older patients, indicating the possibility of an increase over time.^8^^,^^9^

OSL is a multifactorial disease that includes genetic predisposition. Many associated genes and etiological factors have been reported, including age, BMI, hypertension, local mechanical factors, inflammation, hormonal abnormalities, abnormal calcium metabolism, and diabetes, and it is also well known that the degree and speed of progression of ossification vary widely among individuals.^10^^,^^11^ There have also been many reports of biomarkers associated with OSL, but none have been reproducible, and definitive genetic factors and biomarkers have yet to be identified.^12^ Therefore, there are still no therapeutic targets for inhibition of the development and progression of ossification. A common problem in these studies has been the large interindividual variability among the human OSL specimens, and this may be the most significant factor leading to poor reproducibility.

With this background, the focus of this study is on exosomes secreted from cultured cells derived from patients with OSL, which differs from previous analyses. Exosomes are extracellular vesicles (EVs) that are secreted by cells and contain proteins and nucleic acids that signal to surrounding cells; thus, exosomes play important roles in development and progression of diseases. Previous studies indicated that cultured cells derived from the OLF patients exhibited a greater osteogenic differentiation potential than cells cultured from non-OLF patients.^13^^,^^14^ Exosomes secreted by OSL-derived cultured cells are thought to differ from those secreted in patients without OSL, and to contain factors that induce osteogenic differentiation. We hypothesized that exosome membrane proteins derived from OSL cases are involved in development and progression of ossification, and may be indicators of disease (ossification) activity. Exosome analysis is a novel technology that is mainly used in oncology to explore disease development and metastasis, biomarkers, and novel therapeutic targets. Exosome analysis has not been used previously to examine the pathogenesis of OSL. Thus, the aim of this study was to investigate the effect on osteogenic differentiation of exosomes secreted by cultured cells derived from patients with OSL, and to analyze the exosomes involved in development and progression of OSL.

## Materials and methods

### Samples from patients

**Figure 1:**
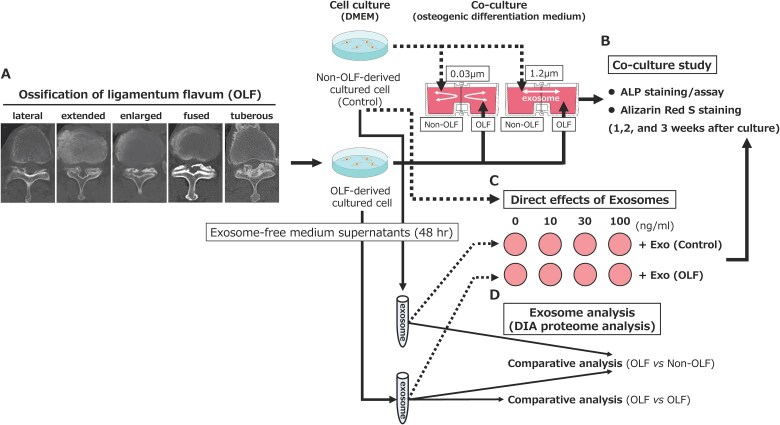
Summary of the study design. (A) Computed tomography classification of thoracic ossification of the ligamentum flavum. (B) A co-culture study was performed to evaluate the effects of exosomes on osteogenic differentiation. (C) Dose-dependent assays were performed to evaluate the direct effects of exosomes on osteogenic differentiation. (D) Comparative exosome analysis (DIA proteome analysis) was performed to detect candidate proteins regulating disease activity of ossification of the ligamentum flavum.

The samples used in the study are listed in Table 1. Ligament-ossified plaque complex samples were collected during posterior decompressive surgery in 22 patients with thoracic OLF (16 males and 6 females; mean age 69.7 yr). Details of these surgeries are given elsewhere.^15^ OLF was identified by the presence of bone growth in the ligamentum flavum on computed tomography (CT) scans of the spine. None of the patients exhibited any signs of congenital bone or joint disorders or abnormalities in musculoligamentous tissue, and none had seronegative spondyloarthropathy or hyperparathyroidism, or were undergoing treatment with glucocorticoids or immunosuppressants. Using a previously described CT classification of thoracic OLF, the lesions were categorized as lateral, extended, enlarged, fused, or tuberous types (Figure 1A). Samples were also obtained from 14 patients without ossification in the whole spine (non-OLF) who underwent lumbar posterior decompressive surgery (10 males and 4 females; mean age 71.1 yr) for use as controls. Patients in the OLF and non-OLF groups were matched based on key demographic and clinical factors, including age, sex, BMI, and comorbidities such as diabetes and hypertension. While we aimed for an age difference within ±3 yr to minimize age-related variability, in some cases, a difference of up to ±7 yr was allowed due to the availability of appropriate matches. The matching process prioritized age, followed by sex, and then BMI (within ±2 kg/m^2^) and comorbidity status (Table 1). Before surgery, written informed consent was obtained from each patient. The Human Ethics Review Committee of the University Medical Faculty approved the study protocol (Approval Number 20220210, 20140046), and the research followed the Clinical Research Guidelines established by the Ministry of Health, Labor, and Welfare of the Japanese Government.

**Table 1:** Demographic data for patients in the study.

**Study**		**Sample Pair**	**OLF cases**						**Non-OLF cases**					
			**Type of OLF**	**Age (yr)/Sex**	**BMI**	**Segment**	**HT**	**DM**	**Diagnosis**	**Age (yr)/Sex**	**BMI**	**Segment**	**HT**	**DM**
**In vitro study/WB**	Co-culture/WB	1	Extended	56/Male	23.6	T10-11	+	−	LSCS	63/Male	24.6	L4-5	+	−
		2	Enlarged	59/Male	24.3	T10-11	−	−	LSCS	63/Male	25.1	L4-5	−	−
		3	Enlarged	75/Male	20.9	T10-11	+	+	LSCS	75/Male	22.8	L4-5	+	−
		4	Enlarged	71/Female	22.3	T11-12	−	−	LSCS	68/Female	20.7	L4-5	−	−
		5	Fused	66/Male	24.3	T9-10	+	−	LSCS	65/Male	25.8	L4-5	+	+
	Direct	1	Enlarged	74/Male	20.8	T12-L1	−	−	LSCS	73/Male	21.4	L4-5	−	−
		2	Fused	70/Female	20.7	T10-11	+	+	LSCS	75/Female	19.7	L4-5	+	−
		3	Enlarged	80/Male	18.9	T10-11	+	−	LSCS	74/Male	19.0	L3-4	+	−
**Exosome analysis OLF vs. Non-OLF**		1	Extended	76/Male	20.9	T10-11	+	−	LSCS	73/Male	22.1	L4-5	+	−
		2	Extended	71/Female	19.7	T10-11	−	−	LSCS	74/Female	18.7	L3-4	−	−
		3	Enlarged	77/Male	22.3	T9-10	+	−	LSCS	74/Male	23,7	L4-5	+	−
		4	Fused	64/Male	23.8	T11-12	−	+	LSCS	71/Male	25.2	L4-5	+	−
		5	Fused	76/Male	21.9	T11-12	+	−	LSCS	80/Male	24.6	L4-5	+	+
		6	Fused	65/Female	24.8	T10-11	−	+	LSCS	67/Female	25.3	L3-4	+	+
**Exosome analysis OLF vs. OLF**		**Sample Pair**	**OLF cases**											
			**Type of OLF**	**Age (yr)/Sex**	**BMI**	**Segment**	**HT**	**DM**	**Type of OLF**	**Age (yr)/Sex**	**BMI**	**Segment**	**HT**	**DM**
		1	Extended	59/Male	24.1	T11-12	−	−	Lateral	58/Male	25.3	T10-11	+	−
		2	Enlarged	71/Male	21.0	T11-12	+	−	Lateral	74/Male	23.3	T10-11	+	−
		3	Fused	80/Female	23.9	T10-11	+	+	Lateral	78/Female	20.8	T10-11	+	+
		4	Tuberous	66/Male	24.1	T11-12	+	−	Lateral	67/Female	24.0	T10-11	−	−

Abbreviations: DM, diabetes; HT, hypertension; OLF, ossification of ligamentum flavum; WB, western blot.

### Cell culture

Ligaments were obtained in a sterile manner from ossified tissue and placed in 100-mm dishes with 20 mL of DMEM (Low Glucose, FUJIFILM Wako Pure Chemical Corp., #041-29 775) containing 10% FBS (GIBCO, #10437028), 0.5% gentamicin (GIBCO, #15710064), and 0.25% amphotericin B (GIBCO, #15290018). Incubation was performed at 37 °C in a humidified atmosphere of 95% air and 5% CO_2_. The cultures were left undisturbed for 2-3 d and then the medium was replaced with an equal volume of fresh medium. Cells obtained from the explants were collected from the dishes using 0.02% EDTA and 0.05% trypsin for further passages, and used in experiments after the third passage.

### Horizontal co-culture

Cultured OLF- and non-OLF-derived cells were placed in a horizontal co-culture system (NICO-1; Ginrei Lab)^16^ at a density of 1 × 10^4^ cells with filters of 0.03 μm (which exosomes do not penetrate) or 1.2 μm (which exosomes penetrate) placed between the wells to evaluate the effect of EVs derived from OLF-derived cultured cells on osteogenic differentiation (*n* = 5 pairs, Table 1 and Figure 1B). Osteogenic differentiation was assessed after 1, 2, and 3 wk of co-culture in osteogenic differentiation medium (1 nM dexamethasone, 50 μg/mL ascorbic acid, and 10 mM β-glycerophosphate) in a humidified atmosphere of 95% air and 5% CO_2_ at 37 °C.

### Assessments of in vitro osteogenic differentiation

To evaluate the osteogenic differentiation of cultured cells, alkaline phosphatase (ALP) activity was evaluated at wk 1, 2, and 3 using an ALP staining kit (Abcam, ab242286) and ALP assay kit (Abcam, ab83369) and standardized to the total protein content in the lysate according to the manufacturer’s instructions. Mineral deposits on the cultured cells were visualized at wk 2 and 3 using an Alizarin Red S staining kit (Bio Future Technologies, BMK-009). The relative expression was quantified at an optical density of 405/650 nm. These experiments were performed once in duplicate for each sample.

### Immunoblot analysis

For immunoblot analysis, the cultured cells (sample pairs 3-5, Table 1) were harvested and solubilized in RIPA lysis buffer 1× (50 mM Tris–HCl, pH 7.4, 150 mM NaCl, 1% NP-40, 0.1% SDS, 0.5% sodium deoxycholate). To determine the protein concentration, we used the DC protein assay kit (Bio-Rad Laboratories) according to Lowry protein Assay. The total protein (10 μg per well) was separated on 12.5% SDS-PAGE and transferred onto polyvinylidene difluoride membrane (PE Applied Biosystems) for 70 min. The membranes were washed twice in PBS solution containing 0.05% Tween 20, then blocked by a mixture of 5% skimmed milk in PBS for 1 h at room temperature, and finally incubated overnight with one of the following antibodies at 4 °C: rabbit anti-Runx2 polyclonal Ab (dilution, 1:250, Proteintech) and rabbit anti-Wnt3a polyclonal Ab (dilution, 1:250, Proteintech) to assess the osteogenic differentiation, and rabbit anti-COLIVA1 polyclonal Ab (dilution, 1:250, GeneTex) rabbit anti-FMNL3 polyclonal Ab (dilution, 1:250, Proteintech), rabbit anti-mTOR polyclonal Ab (dilution, 1:250, Proteintech), or rabbit anti-PIP4K2B polyclonal Ab (dilution, 1:250, Proteintech) to confirm the differential expression of candidate proteins. After washing 3 times in 0.1 M PBS, the membranes were incubated for 1 hr in anti-rabbit secondary IgG/ HRP complex (dilution, 1:5000) (Santa Cruz Biotechnology). After washing 3 times in 0.1 M PBS, the membranes were immersed in Western Lightning ECL Pro (PerkinElmer, CT) for 1 min and examined with imaging analyzer (Fusion Solo.7S. Edge v070, Vilber Bio Imaging). The intensity of each band was quantified using ImageQuantTL (Global Life Sciences Technologies Japan K.K.) and expressed relative to that of β-actin (dilution, 1:1000, Proteintech). For molecular weight controls, we used the ExcelBand 3-color High Range Protein Marker (SMOBIO Thechnology, Inc).

### Isolation and quantification of cultured cell-derived exosomes

For exosome preparation, growing 80% confluent cells were cultured in exosome-free medium for 48 h, after which 300 mL of culture medium was collected. After centrifugation to remove dead cells and cell debris, the supernatant was filtered using a 0.2 μm asymmetric polyether sulfone membrane filter and ultracentrifuged at 3665 rad/s (35 000 rpm) for 70 min at 4 °C. Exosomes congregating at the bottom of the tube were washed and resuspended in PBS. The presence of exosomes was confirmed using ELISA for the exosome markers CD9/CD9 and CD63/CD63 (Hakarel Ltd.). Quantification was performed using 25 μL of isolated exosomes diluted in 200 μL of Protein Assay BCA Kit (FUJIFILM Wako Pure Chemical Corp.; Figure 1C and D).

### Dose-dependent exosome assays on cultured cells

The direct effects of OLF-derived-exosomes on non-OLF-cultured cells were evaluated using dose-dependent assays. Non-OLF-derived cultured cells were seeded in 48-well and 96-well plates and treated with OLF-derived or non-OLF-derived exosomes at concentrations of 0, 10, 30, and 100 ng/mL. This concentration was established because this study found that exosomes isolated from 300 mL of OLF and non-OLF supernatants yielded concentrations 2-15 μg (ie, 6.7-50 ng/mL). Cultured cells were incubated for 1, 2, and 3 wk to assess dose- and time-dependent effects. Osteogenic differentiation was evaluated using ALP activity/assay and Alizarin Red S staining/assays, as previously described (*n* = 3, Figure 1C).

### Sample preparation for proteome analysis

Exosome-free cell supernatants (300 mL) were desalted and dried using a centrifugal evaporator. Protein dissolution was achieved using a sealed ultrasonic disruption machine in a solution containing 100 mM Tris (pH 8.0), 4% SDS, and 20 mM NaCl. The protein concentration was measured using the bicinchonic acid assay (BCA) assay and adjusted to 0.2 μg/μL with the same buffer. To cleave the S-S bonds of the protein, tris(2-carboxyethyl)phosphine (TCEP) was added to the protein lysate at a final concentration of 20 mM and incubated at 80 °C for 10 min. To alkylate the cysteine residues, iodoacetamide was added at a final concentration of 30 mM and incubated at room temperature (in the dark) for 30 min. Two types of beads, Sera-Mag SpeedBead carboxylate-modified magnetic particles (Hydrophobic) and Sera-Mag carboxylate-modified magnetic particles (Hydrophobic) (Cytiva), were used for single-pot solid-phase-enhanced sample preparation (SP3 method). These beads were combined at a 1:1 vol:vol ratio and washed 3 times with distilled water to achieve a concentration of 8 μg solids/μL (SP3 beads). Subsequently, 20 μL of SP3 beads was added to the alkylated sample and an equal volume of ethanol was added. The mixture was then stirred at room temperature for 20 min. The beads were washed twice with 80% ethanol and mixed with 100 μL of 50 mM Tris–HCl (pH 8.0). To break down the proteins into smaller peptide fragments, 500 ng Trypsin/Lys-C Mix (Promega K.K.) was added and incubated overnight at 37 °C. A volume of 20 μL of 5% trifluoroacetic acid (TFA) was added, and the mixture was processed in a sample-tight sonication machine and then applied to a reverse-phase spin column (GL-Tip SDB, GL Science). After desalting, the samples were dried in a centrifugal evaporator. Ultrasonically disrupted peptides were dissolved in 2% acetonitrile (CAN)-0.1% TFA, and the peptide concentration was measured by BCA assay, adjusted with 2% ACN-0.1% TFA to 200 ng/μL peptide, and used for LC–MS analysis.

### Data-independent acquisition proteome analysis

Peptides were measured using nano liquid chromatography–tandem mass spectrometry (on an UltiMate 3000 RSLCnano LC system (Thermo Fisher Scientific). A 75 μm × 120 mm column with a Q Exactive HF-X (Thermo Fisher Scientific) was used for overlapping window data-independent acquisition (DIA)-MS. MS1 spectra were collected in the range of 495-745 *m*/*z* at 30 000 resolution with an automatic gain control target of 3 × 10^6^ ions and a maximum injection time of 55 ms. MS2 spectra were gathered at a mass-to-charge ratio > 200, a resolution of 30 000, and an automatic gain control target of 3 × 10^6^ ions. The maximum injection time was set to auto and the stepped normalized collision energy was set to 23%. The isolation width for MS2 was set at 6.0 *m*/*z*. Overlapping window patterns of 500-740 *m*/*z* were utilized for window placements. Data obtained from MS were analyzed using Scaffold DIA software (Proteome Software). The predicted human spectral library was generated using the Human Protein Sequence Database (Proteome ID UP000005640). Protein identities were determined by mapping peptides to their respective proteins during the protein inference process. The search parameters used for the experimental data were as follows: trypsin as the enzyme for the search, a maximum of one missed cleavage site, precursor mass tolerance of 10 ppm, fragment mass tolerance of 10 ppm, and cysteine carbamidomethylation. The protein identification threshold was set at <1% for both peptide and protein false discovery rates. Protein quantification was calculated using DIA-NN software (v. 1.8.1), providing a comprehensive analysis pipeline including protein abundance estimation. For protein quantification, abundances were derived by summing peptide intensities. Protein abundance data were normalized using quantile normalization to correct for technical variability across samples. This analytical workflow accurately quantifies proteins by aligning peptide data and applying normalization to improve inter-sample comparisons.

### Analysis of molecular expression, networks, and pathways

Using the results of DIA proteome analysis, the fold change (FC) of proteins (OLF-derived exosomes *vs.* non-OLF-derived exosomes) was calculated. Proteins with FC >2.0 in 3 or more of the 6 pairs were considered to be increased, and those with FC <0.5 as decreased. Similarly, FC between lateral-type OLF and each OLF type (extended, enlarged, fused, and tuberous types) was calculated to compare peptide expression according to the degree of ossification. The molecular networks and pathways associated with increased and decreased factors were analyzed using KeyMolnet Viewer (ver. 6.2, content ver. 9.7.20230927165918, KM Data Inc.). KeyMolnet is a commercial knowledge base that includes over 260 000 manually curated relationships between human genes and proteins, small molecules, diseases, pathways, and drugs. This knowledge base includes core content that has been carefully selected from review articles with the highest level of reliability.^17^ Molecular network analysis was carried out using the “start points and endpoints” search algorithm to identify the network through which differentially expressed molecules influence target molecules. This analysis focused on the upstream and downstream networks of the targets. In assessment of pathways involved in the networks, the similarity between the extracted network and canonical pathway was defined by the HScore, which was calculated based on a hypergeometric distribution formula.^18^ An HScore ≥20 was considered to be significant.^19^

### Statistical analysis

Data are shown as mean ± SD. Categorical variables were compared by Mann–Whitney U test or chi-square test, with a significance level of *p* <.05. EZR (Saitama Medical Center, Jichi Medical University), a graphical user interface for R (The R Foundation for Statistical Computing), was used for all analyses.^20^

## Results

### Effects on osteogenic differentiation of exosomes from spinal ligament-derived cells

**Figure 2:**
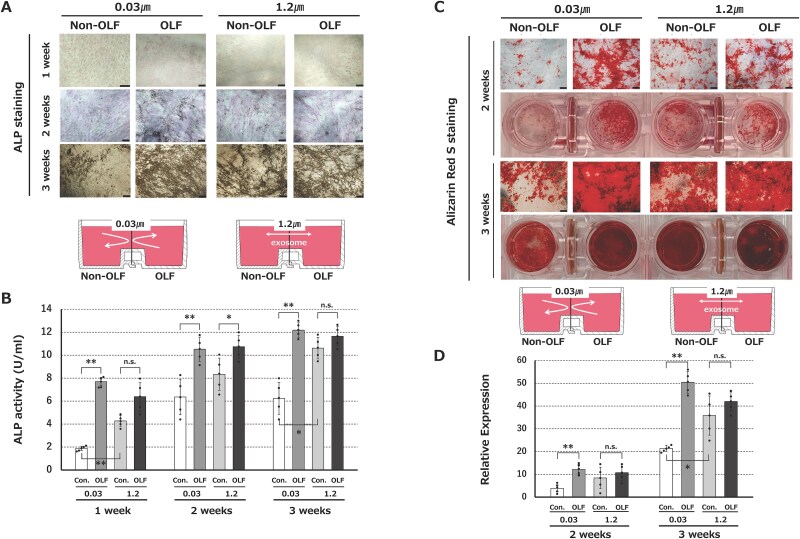
(A) ALP staining at weeks 1, 2 and 3 after culture in osteogenic differentiation medium using exosome-penetrating filters (1.2 μm) and exosome-non-penetrating filters (0.03 μm). (B) Changes in ALP activity of OLF-derived and non-OLF-derived (control) cells in co-culture with 0.03- and 1.2-μm filters. (C) Alizarin Red S staining at wk 2 and 3 after culture in osteogenic differentiation medium using exosome-penetrating (1.2 μm) and exosome-non-penetrating (0.03 μm) filters. (D) Changes in relative expression of OLF-derived and non-OLF-derived (control) cells in co-culture with 0.03- and 1.2-μm filters. Data are expressed as mean ± SD. Scale bar = 200 μm. ^*^p<.05, ^**^p<.01.

In horizontal co-culture of non-OLF-derived cells with OLF-derived cells, use of 1.2-μm filters (which exosomes penetrate) resulted in significantly higher osteogenic differentiation of the non-OLF cells assessed by ALP staining and in assays at wk 3, compared to use of 0.03-μm filters (which exosomes do not penetrate). ALP activity in non-OLF-derived cells did not differ significantly from that in OLF-derived cells at 3 wk after culture using 1.2-μm filters in osteogenic differentiation medium, but there were significant differences between the 2 cell types in co-culture with 0.03-μm filters (Figure 2A and B). Calcium deposits assessed by Alizarin Red S staining in non-OLF cells co-cultured with OLF cells significantly increased using 1.2-μm filters compared to 0.03-μm filters at 3 wk after culture. Relative expression of non-OLF-derived cells did not differ significantly from that of OLF-derived cells at 3 wk after culture in osteogenic differentiation medium, whereas there were significant differences between the cell types in co-culture with 0.03-μm filters (Figure 2C and D).

**Figure 3:**
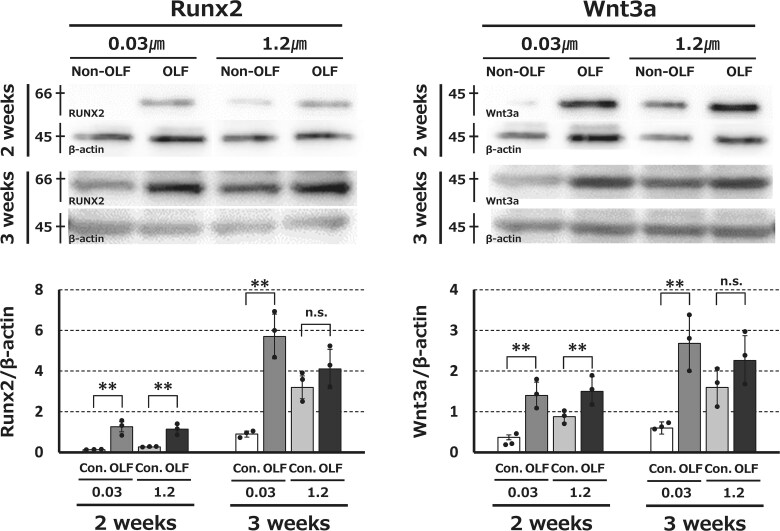
(A) Western blots revealing Runx2 and Wnt3a expression at wk 2 and 3 following culture in osteogenic differentiation medium using exosome-penetrating (1.2 μm) and exosome-non-penetrating (0.03 μm) filters. (B) Expression levels of Runx2 and Wnt3a of OLF-derived and non-OLF-derived (control) cells co-cultured with 0.03- and 1.2-μm filters. (*n* = 3 each). ^**^p<.01.

Western blot analysis revealed time-dependent changes in the osteogenic differentiation markers Runx2 and Wnt3a (Figure 3). Runx2 and Wnt3a expression differed significantly between OLF-derived and non-OLF-derived cells in co-culture with 0.03-μm filters at 2 and 3 wk after culture. At 3 wk of co-culture with 1.2-μm filters, neither group showed significant differences.

### Direct effects of OLF-derived exosomes on non-OLF-cultured cells

**Figure 4:**
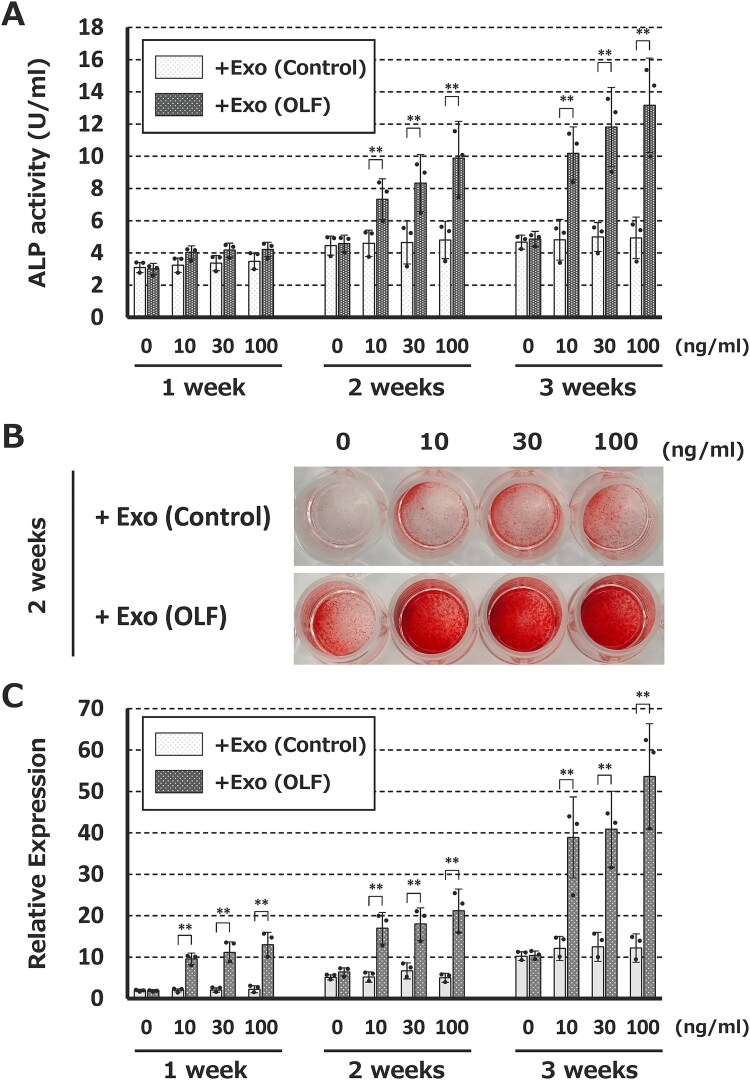
Direct effects of OLF-derived exosomes on non-OLF-cultured cells. Cultured cells treated with OLF-derived exosomes (Exo (OLF)) exhibited significant dose-dependent (0, 10, 30, and 100 ng/mL) increases in osteogenic differentiation, whereas the addition of non-OLF-derived exosomes (Exo (control)) did not affect osteogenic differentiation. (A) Alkaline phosphatase (ALP) assay. (B) Alizarin Red S staining. (C) Relative expression assay of Alizarin Red S.

Dose-dependent OLF-derived exosome assays on non-OLF-derived cultured cells showed a dose-dependent increase in osteogenic differentiation, as assessed by ALP staining/assay and Alizarin Red S staining/assay. Cultured supplemented with non-OLF-derived exosomes showed no significant dose-dependent changes (Figure 4).

### Comparative proteome analysis of OLF- and non-OLF-derived exosomes

**Figure 5:**
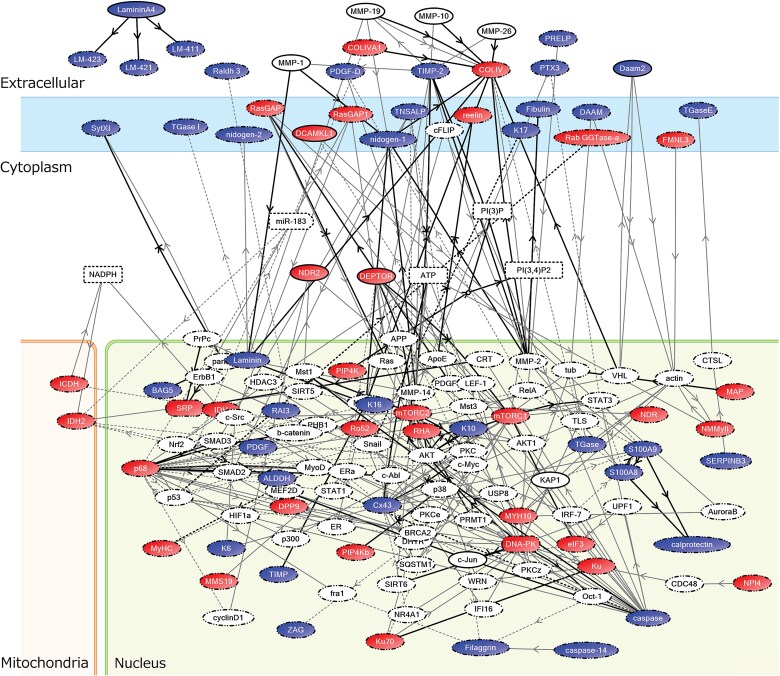
The “start points and endpoints” network search algorithm in KeyMolnet produced an intricate network of targets with significant associations. Nodes with higher expression in OLF-derived exosomes than in non-OLF-derived exosomes are highlighted, and nodes with lower expression in OLF-derived exosomes than in non-OLF-derived exosomes are also marked. Molecular relationships are indicated by solid lines with an arrow (direct binding or activation) and without an arrow (complex formation).

Exosomes with protein concentrations of 2-15 μg/100 μL were isolated from supernatants of OLF- and non-OLF-derived cultured cells. The amount of isolated exosomes was higher in OLF-derived exosomes than in non-OLF-derived exosomes and higher in samples from OLF cases with a greater degree of ossification. In comparative proteome analysis of OLF and non-OLF-derived exosomes, more than 3000-5000 proteins were identified per sample pair using DIA-NN software (v. 1.8.1), and 32 upregulated factors (FC >2) and 40 downregulated factors (FC <0.5) were identified (Tables S1 and S2). To identify the relationships between the molecular network of the exosome proteome and pathways, the 72 differentially expressed proteins were imported into KeyMolnet and an interrelated network search was performed (Figure 5). In the extracted molecular network, 10 pathways with HScored >20 and contributed significantly to the network (Table 2).

**Table 2:** Top 10 pathways contributing to the interrelated network of candidate proteins for regulation of ossification calculated by KeyMolnet.

**Rank**	**Pathway**	**Score**
**1**	Autophagy-related protein signaling pathway	61.737
**2**	AKT signaling pathway	52.128
**3**	Transcriptional regulation by SMAD	50.446
**4**	Transcriptional regulation by AP-1	49.991
**5**	MMP signaling pathway	48.389
**6**	mTOR signaling pathway	46.550
**7**	Transcriptional regulation by STAT	43.519
**8**	Sirtuin signaling pathway	40.777
**9**	Wnt signaling pathway	39.263
**10**	Transcriptional regulation by VDR	37.285

### Fold change of peptides based on the degree of ossification

Of the 32 upregulated factors, 17 were identified as exosome membrane proteins. Of the 40 downregulated factors, 30 were identified as exosome membrane proteins. The FCs of these factors based on the degree of ossification (lateral type *vs*. extended, enlarge, fused, and tuberous types) are shown in Tables 3 and 4. COLIVA1, Formin-like (FMNL) protein 3, mTORC2 and PIP4K were identified as factors that satisfied 3 conditions in OLF-derived exosomes: (1) upregulated compared with non-OLF-derived exosomes, (2) exosome membrane proteins, and (3) upregulated based on the degree of ossification. Fatty acid-binding protein (FABP) 5, Keratin (KRT) family proteins (Intermediated filament [IF]-I [KRT10, 14, 16, and 17] and IF-II [KRT2, 6A, 6B, and 80]), S100A8, Serpin (SERPIN) B3, and Transglutaminase (TGM) were identified as factors that satisfied 3 conditions in OLF-derived exosomes: (1) downregulated compared with non-OLF-derived exosomes, (2) exosome membrane proteins, and (3) downregulated based on the degree of ossification.

**Table 3:** Fold changes of candidate upregulated proteins between patients with each type of OLF and those with lateral type OLF.

**Symbol**	**Protein name**	**Fold changes of proteins in EVs of patients with OLF**			
		**Extended/lateral** **(Sample Pair 1)**	**Enlarged/lateral** **(Sample Pair 2)**	**Fused/lateral** **(Sample Pair 3)**	**Tuberous/lateral** **(Sample Pair 4)**
**PSMD8**	26S proteasome non-ATPase regulatory subunit 8	0.736	2.905	0.380	0.280
**COL4A1**	Collagen alpha-1(IV) chain	N/A	6.786	6.876	6.127
**DCLK1**	Serine/threonine-protein kinase DCLK1	0.312	0.682	1.908	0.596
**XRCC6**	X-ray repair cross-complementing protein 6	0.671	0.386	1.310	0.879
**DHX9**	ATP-dependent RNA helicase A	0.470	0.296	0.892	0.419
**EPS8L2**	Epidermal growth factor receptor kinase substrate 8-like protein 2	1.956	1.031	0.868	1.698
**FMNL3**	Formin-like protein 3	1.911	1.937	1.639	3.132
**HSPE1**	10 kDa heat shock protein, mitochondrial	0.543	0.923	0.601	0.326
**MDH2**	Malate dehydrogenase, mitochondrial	0.738	0.803	0.453	0.335
**mTORC1**	Serine/threonine-protein kinase mammalian TOR complex 1	1.003	1.211	0.942	0.921
**mTORC2**	Serine/threonine-protein kinase mammalian TOR complex 2	1.185	1.224	1.196	1.156
**MYH10**	Myosin heavy chain 10	1.112	0.709	0.577	0.641
**NBEAL2**	Neurobeachin-like protein 2	0.268	3.377	3.444	0.923
**PIP4K2B**	Phosphatidylinositol 5-phosphate 4-kinase type-2 beta	0.968	1.008	3.092	2.992
**RABGGTA**	Geranylgeranyl transferase type-2 subunit alpha	1.236	1.091	0.645	0.797
**RAB3GAP1**	Rab3 GTPase-activating protein catalytic subunit	1.069	1.096	0.966	1.033
**RASA1**	Ras GTPase-activating protein 1	1.587	0.969	1.632	2.590

Abbreviations: EV, extracellular vesicles; OLF, ossification of the ligamentum flavum. Bold symbols indicate upregulated exosomal membrane proteins in OLF samples with greater ossification. Shaded cells represent proteins with increased expression relative to the lateral type.

**Table 4:** Fold changes of candidate downregulated proteins between patients with each type of OLF and those with lateral type OLF.

**Symbol**	**Protein name**	**Fold changes of proteins in EVs of patients with OLF**			
		**Extended/lateral** **(Sample Pair 1)**	**Enlarged/lateral** **(Sample Pair 2)**	**Fused/lateral** **(Sample Pair 3)**	**Tuberous/lateral** **(Sample Pair 4)**
**ALDH1A3**	Retinaldehyde dehydrogenase 3	0.15860971	1.026849	2.26939093	0.359947437
**CASP14**	Caspase-14	1.031941881	0.119078	0.447859255	0.462164722
**GJA1**	Gap junction alpha-1 protein	2.524111679	1.852408	1.002472018	2.530351328
**CRIM1**	Cysteine-rich motor neuron 1 protein	1.27032039	3.167027	4.839392934	6.14757952
**DSP**	Desmoplakin	1.135730751	0.05449	0.181906734	0.206597072
**FABP5**	Fatty acid-binding protein 5	0.93730773	0.205044	0.544393056	0.510263819
**HMCN1**	Hemicentin-1	7.355477319	13.98797	0.938330654	6.901869839
**SLC2A5**	Solute carrier family 2, facilitated glucose transporter member 5	1.054063665	0.181011	2.043746355	2.154238773
**KRT14**	Keratin, type I cytoskeletal 14	0.603428668	0.08236	0.27791852	0.167704002
**KRT16**	Keratin, type I cytoskeletal 16	0.459683265	0.065839	0.394254053	0.18123199
**KRT10**	Keratin, type I cytoskeletal 10	0.80271977	0.13969	0.308186481	0.247387381
**KRT17**	Keratin, type I cytoskeletal 17	0.305128697	0.073289	0.44268052	0.13507453
**KRT6A**	Keratin, type II cytoskeletal 6A	0.444035992	0.123351	0.336540368	0.149436037
**KRT6B**	Keratin, type II cytoskeletal 6B	0.532292019	0.110105	0.307918639	0.163902634
**KRT2**	Keratin, type II cytoskeletal 2 epidermal	0.62166421	0.111127	0.29281148	0.182030418
**KRT80**	Keratin, type II cytoskeletal 80	0.919946198	0.063454	0.137365792	0.126369138
**LAMA4**	Laminin subunit alpha-4	0.277682871	3.574759	2.539378635	0.705141951
**MELTF**	Melanotransferrin	1.311231926	1.462242	0.256983558	0.336965046
**NID1**	Nidogen-1	0.143176443	4.166825	4.821832095	0.690372769
**NID2**	Nidogen-2	0.070742912	16.58719	25.15373022	1.779448118
**GPRC5A**	Retinoic acid-induced protein 3	1.597081238	1.988698	2.100453466	3.354594822
**S100A8**	Protein S100-A8	0.766402139	0.027947	0.78107552	0.598617949
**SERPINB3**	Serpin B3	0.601559089	0.044849	0.40804377	0.245462438
**SYT11**	Synaptotagmin-11	1.279543103	2.986281	3.49003414	4.465649113
**TNC**	Tenascin-C	0.178641014	1.071303	4.364896627	0.779749561
**TGM1**	Protein-glutamine gamma-glutamyltransferase K	0.959384924	0.073197	0.210485038	0.201936172
**TGM3**	Protein-glutamine gamma-glutamyltransferase E	0.754830487	0.036889	0.243100342	0.18349955
**TMEM59**	Transmembrane protein 59	0.862064518	2.519768	3.410389412	2.939975703
**ALPL**	Alkaline phosphatase, tissue-nonspecific isozyme	0.044275885	1.582206	18.84789965	0.834507441
**AZGP1**	Zinc-alpha-2-glycoprotein	1.340309434	0.171849	0.359364564	0.481659715

Abbreviations: EV, extracellular vesicles; OLF, ossification of the ligamentum flavum. Bold symbols indicate downregulated exosomal membrane proteins in OLF samples with greater ossification. Shaded cells represent proteins with decreased expression relative to the lateral type.

### Western blot analysis of candidate proteins

**Figure 6:**
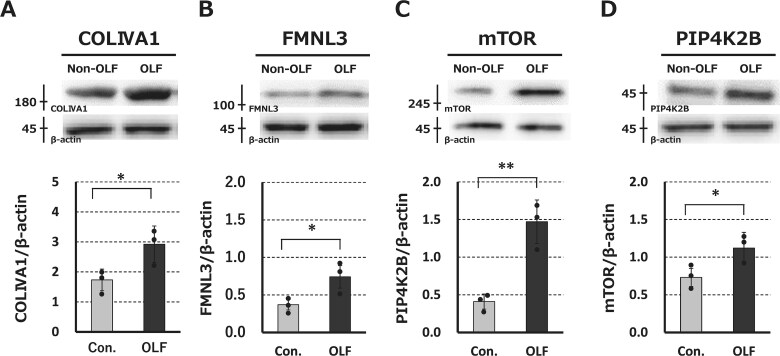
Western blots illustrating baseline expression differences in COLIVA1 (A), FMNL3 (B), mTOR (C), and PIP4K2B (D) between OLF- and non-OLF (control)-derived cultured cells. ^*^p<.05, ^**^p<.01.

Western blotting was performed to further confirm the differential expression of candidate proteins involved in the progression of ossification obtained from proteome analysis (*n* = 3 pairs). Expression of COLIVA1, FMNL3, mTOR, and PIP4K2B were significantly higher in OLF-derived cultured cells than in non-OLF (Figure 6).

## Discussion

This study investigated the role of exosomes derived from ossified ligament cells (OLF-derived exosomes) in osteogenic differentiation and identified candidate regulatory factors through proteomic analysis. Our findings offer novel insights into the underlying molecular mechanisms of OSL. OSL includes OPLL and OLF, and is a multifactorial disease for which many candidate genes and biomarkers have been reported.^11^^,^^12^ However, there are no definitive and quantitative conclusions to date, probably because of low reproducibility due to individual differences in human OSL specimens and blood data used in previous studies.

In a horizontal co-culture study, non-OLF-derived cells co-cultured with OLF-derived cells using exosome-penetrating 1.2-μm filters had significantly greater osteogenic differentiation, as assessed by ALP, Alizarin Red S staining, and western blotting of Runx2 and Wnt3a, compared to co-culture using 0.03 μm filters, which exosomes do not penetrate. These results support the hypothesis that OLS-derived exosomes are involved in disease activity of osteogenic differentiation. Furthermore, the dose-dependent increases in ALP activity and mineral deposition in non-OLF cells treated with OLF-derived exosomes further confirmed their osteoinductive potential. Comparative proteomics analysis of OLF and non-OLF-derived exosomes identified 32 upregulated and 40 downregulated factors that were in an interrelated network involved in regulation of ossification, including the MMP signaling, mTOR signaling, Wnt signaling and VDR-associated pathways. Among the exosomal membrane proteins upregulated in OLF samples, COL IV, FMNL3, mTORC2, and PIP4K had increased expression with greater ossification, and thus, may be candidate regulators of OSL.

Histologically, the process of ossifying extension in OSL is similar to enchondral ossification, which occurs in the growth plates of long bones.^21^ We found that OPLL and OLF ossification involve chondrocyte differentiation, characterized by unique expression of various transcriptional factors. Additionally, differentiation of hypertrophic chondrocytes around the calcified area of the ossification front is closely associated with the process of ossification.^13^^,^^22^ COLIVA1, FMNL3, mTORC2, and PIP4K, which were identified as candidate regulators of OSL in this study, are involved in the PI3K/Akt/mTOR signaling pathway. mTOR regulates chondrocyte differentiation via interactions with SOX9, RUNX2 and Indian hedgehog (Ihh).^23^ Upregulation of FMNL3, an actin cytoskeletal remodeling regulator, further supports its involvement in ossification progression. Inducing Ihh with SOX9 or PTHrP promotes differentiation and maturation of chondrocytes during the early stage of normal enchondral ossification. Additionally, Ihh stimulates calcification through RUNX2 in the late stage by controlling the maturation of chondrocytes.^24^^,^^25^ In our previous study, we observed significantly elevated expression of Ihh, PTHrP, and SOX9 in cultured cells from patients with OPLL compared to those from non-OPLL patients.^26^^,^^27^ mTOR complex, mTORC2, interacts with Wnt and IGF-2 and affects osteoblast differentiation. A previous study revealed that removal of the *Rictor* gene, which is an essential component of mTORC2, resulted in smaller bones in mouse limbs.^28^ Another study showed that RUNX2 activates PI3K/Akt signaling through mTORC2 in breast cancer cells.^29^ In our previous study using OLF samples, OLF cells subjected to cyclic tensile strain showed activation of ossification properties mediated by Wnt/β-catenin signaling.^14^ Additionally, the candidate gene highlighted by the genome-wide association study analysis, RSPO2, functions as a regulator of the Wnt/β-catenin signaling pathway.^30^ Thus, it can be inferred that mTOR is likely to be involved in OSL through these mechanisms. PIP4K, a regulator of mTOR signaling, and PIP4K2B significantly influence cell proliferation. Several recent reports have shown an association between PIP4K and cancer cell proliferation,^31^^,^^32^ but a relationship with osteogenic differentiation has not been described and requires further study.

Type IV collagen is a network-forming molecule that is an abundant component of the basement membrane. Type IV collagen is also involved in regulation of cell functions such as cell adhesion, migration, proliferation and differentiation through interactions with cell membrane receptors.^33^ A recent study indicated an association of degradation of type IV collagen with mesenchymal stem cell (MSC) migration and trans-endothelial migration.^34^ There are no reports showing a direct relationship between type IV collagen and OSL, but several studies have suggested that MSCs from OSL samples have higher osteogenic differentiation potential compared with those from non-OSL samples, which indicates that MSCs may be involved in ossification in spinal ligaments.^35–37^ MSC migration may determine the activity of osteogenic differentiation and be crucial for promoting OSL.

In contrast, FABP5, several KRT family proteins, along with S100A8, SERPINB3, and TGM were significantly downregulated in OLF-derived exosomes. FABP5 is known for its role in intracellular lipid transport and metabolism. Recent studies suggest a potential association between FABP5 and bone formation, particularly in endochondral ossification.^38^ KRT participates cellular stress responses and cytoskeletal reorganization, suggesting a role in mesenchymal stem cell migration and ossification during tissue remodeling.^39^ S100A8, a calcium-binding protein, contributes to osteogenesis through its involvement in inflammatory processes. Studies suggest that S100A8 influences osteoclast and osteoblast activity, affecting bone resorption and formation.^40^ SERPINB3, a serine protease inhibitor, regulates inflammation and suppresses apoptosis; its increased expression is linked to fibrosis and tissue hardening, potentially enhancing the inflammatory microenvironment associated with ossification.^41^ TGM catalyze protein cross-linking and stabilize the extracellular matrix (ECM), but their dysregulation may lead to pathological calcification and ECM remodeling.^42^ In this context, the downregulation suggests a potential shift in cellular signaling that favors ossification.

This study has certain limitations. First, cultured cells derived from patients with various types of OLF were used, but the sample size was not sufficient to minimize individual patient differences. Additionally, given the impact of demographic factors such as age, sex, BMI, comorbidities (hypertension and diabetes), and other factors on EVs, it is important to exercise caution when interpreting the results and to recognize that there are variations in ossification activity in each patient. Second, the results of the in vivo and in vitro studies involving human OLF-related samples showed disparities. This indicates that cellular reactions in cell culture may not be applicable to the in vivo scenario of cells in the extracellular matrix. Third, relying primarily on a FC threshold of >2 or <0.5 to identify significant proteins may not fully account for natural variations in protein expression levels, although this approach is commonly accepted in exploratory proteomics studies with limited sample sizes. Fourth, functional validation, such as gain- or loss-of-function studies, was not performed to confirm the proteomic findings of this study. Lastly, this study included thoracic OLF cases as samples of OSL and did not use samples from OPLL cases. Cervical OPLL and thoracic OLF are often viewed as similar conditions,^3^ but differences in patient characteristics have also been reported.^43^ Therefore, the results of the study may not be applicable to OPLL. Despite these limitations, our results provide important insights into the pathophysiology of OSL progression. Candidate exosomal membrane proteins associated with disease activity, identified in this study, represent potential biomarkers and novel therapeutic targets. Future research should validate these findings in larger cohorts and explore the functional roles of the identified proteins using gene editing and in vivo models. These studies could provide deeper insights into OSL pathogenesis and facilitate the development of targeted therapies.

## Conclusion

The etiology and pathogenesis of OSL have attracted considerable attention, and many candidate genetic biomarkers have been identified. However, there are no definitive and quantitative conclusions to date, probably because of low reproducibility due to individual differences in human OSL specimens. Exosomes contain proteins and nucleic acids that signal to surrounding cells, and play an important role in development and progression of diseases. Exosome analysis is a novel technology with potential applications in exploration of disease development, biomarkers, and novel therapeutic targets, mainly in oncology. This is the first study to focus on exosomes secreted by OSL-derived cells, which are considered to be responsible for ectopic ossification of the spinal ligament, and to analyze their possible involvement in development and progression of ossification. In a horizontal co-culture study, non-OLF-derived cells co-cultured with OLF-derived cells with exosome-penetrating 1.2-μm filters had significantly increased osteogenic differentiation compared to use of exosome-non-penetrating 0.03-μm filters. In addition, the dose-dependent increases in osteogenic differentiation in non-OLF cells treated with OLF-derived exosomes were observed. These results strongly suggests that ligament cells derived from patients with OLF undergo transformation, and that OLF-derived exosomes are involved in disease activity of osteogenic differentiation. Comparative proteomic analysis detected 32 increased and 40 decreased factors in OLF-derived exosomes compared to non-OLF-derived exosomes. Among these factors, COL IV, FMNL3, mTORC2 and PIP4K were upregulated exosome membrane proteins with greater ossification, suggesting they may be candidate regulators of OSL. In contrast, several KRT family proteins, S100A8, SERPINB3, and TGM were significantly downregulated in OLF-derived exosomes. Thus, these proteins are candidate regulators of OSL and may serve as biomarkers and therapeutic targets in this disease.

## Supplementary Material

R1_Supplementary_Table_1_ziaf021

R1_Supplementary_Table_2_ziaf021

## Data Availability

Data generated and analyzed during this study are included in this published article. Data and materials are available from the corresponding author subject to reasonable request and subject to the ethical approvals in place and materials transfer agreements.
