# Supplementary material for: Proteomic analysis and effects on osteogenic differentiation of exosomes from patients with ossification of the spinal ligament
Source: JBMR Plus. 2025 Feb 2;9(4):ziaf021. doi: 10.1093/jbmrpl/ziaf021 (PMC11911064; doi:10.1093/jbmrpl/ziaf021)
Supplement: R1_Supplementary_Table_1_ziaf021 [file r1_supplementary_table_1_ziaf021.docx]

**Supplementary Table 1.** Upregurated factors

| **Accession Number** | **Gene Symbol** | **Sample Pair 1** | **Sample Pair 2** | **Sample Pair 3** | **Sample Pair 4** | **Sample Pair 5** | **Sample Pair 6** |
| --- | --- | --- | --- | --- | --- | --- | --- |
| O15075 | DCLK1 | 0.404 | 0.926 | 4.743 | 2.218 | 3.250 | 1.906 |
| O15372 | EIF3H | 0.176 | 1.018 | 3.038 | 2.056 | 2.429 | 1.943 |
| O76094 | SRP72 | 0.622 | 0.903 | 2.904 | 1.353 | 2.177 | 2.422 |
| P00813 | ADA | MV | 6.785 | 37.258 | 0.993 | 2.285 | 0.878 |
| P02462 | COL4A1 | MV | 3.006 | 0.296 | 11.780 | 1.736 | 2.794 |
| P12956 | XRCC6 | 0.696 | 1.089 | 2.673 | 1.070 | 2.773 | 4.418 |
| P20936 | RASA1 | 0.061 | 0.935 | 4.398 | 2.245 | 2.315 | 1.241 |
| P29144 | TPP2 | 0.439 | 1.021 | 2.407 | 2.275 | 2.167 | 1.216 |
| P35573 | AGL | 0.900 | 1.117 | 9.345 | 3.213 | 7.655 | 1.222 |
| P35580 | MYH10 | 1.028 | 2.032 | 3.513 | 0.734 | 1.035 | 2.965 |
| P40926 | MDH2 | 3.012 | 0.838 | 1.394 | 2.270 | 2.826 | 1.980 |
| P48556 | PSMD8 | 0.030 | 1.108 | 2.464 | 3.585 | 1.234 | 2.151 |
| P48735 | IDH2 | MV | 1.020 | 8.527 | 1.502 | 3.978 | 2.266 |
| P61604 | HSPE1 | 2.572 | 0.794 | 1.273 | 2.855 | 3.094 | 1.963 |
| P78356 | PIP4K2B | 2.564 | 1.812 | 0.976 | 3.119 | 3.095 | 1.760 |
| P78509 | RELN | 4.548 | 0.322 | 0.500 | 35.183 | 4.995 | MV |
| Q08211 | DHX9 | 0.683 | 1.318 | 2.134 | 0.875 | 2.957 | 3.627 |
| Q15042 | RAB3GAP1 | 0.679 | 0.994 | 2.672 | 3.014 | 2.749 | 1.397 |
| Q15075 | EEA1 | 3.440 | 1.467 | 5.616 | 1.248 | 3.946 | 1.820 |
| Q15393 | SF3B3 | 1.097 | 1.585 | 2.663 | 0.846 | 2.071 | 4.264 |
| Q6ZNJ1 | NBEAL2 | 9.123 | 0.275 | 0.707 | 7.601 | 2.251 | 1.160 |
| Q86TI2 | DPP9 | 0.418 | 0.912 | 4.186 | 2.377 | 2.122 | 1.044 |
| Q8IVF7 | FMNL3 | 3.345 | 0.992 | 0.615 | 3.945 | 2.037 | 1.428 |
| Q8N6Y2 | LRRC17 | 2.944 | 4.114 | 0.347 | 3.458 | 4.263 | 1.823 |
| Q8TAT6 | NPLOC4 | 0.429 | 1.033 | 2.262 | 2.687 | 3.466 | 1.410 |
| Q8TB45 | DEPTOR (mTORC1, mTORC2) | 0.215 | 2.005 | 49.276 | 4.333 | 8.360 | 1.894 |
| Q92696 | RABGGTA | 0.201 | 0.831 | 3.212 | 2.361 | 2.164 | 1.161 |
| Q96T76 | MMS19 | 0.000 | 1.072 | 6.250 | 2.055 | 2.847 | 1.455 |
| Q9H6S3 | EPS8L2 | 0.299 | 1.033 | 2.488 | 2.084 | 2.021 | 1.111 |
| Q9NTX5 | ECHDC1 | 0.042 | 0.736 | 6.486 | 2.597 | 3.843 | 1.490 |
| Q9UBQ5 | EIF3K | 0.400 | 0.915 | MV | 8.148 | 2.097 | 1.292 |
| Q9Y2H1 | STK38L | 0.605 | 1.615 | 3.749 | 2.324 | 3.738 | 1.638 |
