# Supplementary material for: Proteomic analysis and effects on osteogenic differentiation of exosomes from patients with ossification of the spinal ligament
Source: JBMR Plus. 2025 Feb 2;9(4):ziaf021. doi: 10.1093/jbmrpl/ziaf021 (PMC11911064; doi:10.1093/jbmrpl/ziaf021)
Supplement: R1_Supplementary_Table_2_ziaf021 [file r1_supplementary_table_2_ziaf021.docx]

**Supplementary Table 2.** Downregurated factors

| **Accession Number** | **Gene Symbol** | **Sample Pair 1** | **Sample Pair 2** | **Sample Pair 3** | **Sample Pair 4** | **Sample Pair 5** | **Sample Pair 6** |
| --- | --- | --- | --- | --- | --- | --- | --- |
| P02533 | KRT14 | 0.424 | 7.757 | 0.132 | 0.134 | 1.632 | 0.519 |
| P02538 | KRT6A | 9.088 | 0.016 | 0.076 | 0.149 | 1.210 | 0.189 |
| P04259 | KRT6B | 10.363 | MV | 0.095 | 0.087 | 0.786 | 0.189 |
| P05109 | S100A8 | 21.711 | 0.024 | 0.323 | 0.176 | 6.289 | 0.058 |
| P05186 | ALPL | 1.451 | 0.300 | 0.254 | 0.614 | 0.388 | 0.300 |
| P05452 | CLEC3B | 13.239 | 0.423 | 0.171 | 0.022 | 0.851 | 0.485 |
| P08582 | MELTF | 9.548 | 0.281 | 1.569 | 0.313 | 0.214 | 0.541 |
| P08779 | KRT16 | 10.565 | 0.054 | 0.123 | 0.076 | 1.157 | 0.291 |
| P12273 | PIP | 5.732 | 0.114 | 0.351 | 0.208 | 0.388 | 0.231 |
| P13645 | KRT10 | 11.421 | 0.069 | 0.467 | 0.147 | 1.052 | 0.191 |
| P14543 | NID1 | 0.279 | 5.728 | 0.333 | 1.806 | 0.433 | 0.216 |
| P15924 | DSP | 0.450 | 13.836 | 0.181 | 0.223 | 4.092 | 0.648 |
| P16035 | TIMP2 | 0.342 | 10.582 | 0.387 | 0.244 | 0.901 | 0.230 |
| P17302 | GJA1 | 0.185 | 3.876 | 1.950 | 0.464 | 0.251 | 0.143 |
| P22732 | SLC2A5 | 0.474 | 0.322 | 2.361 | 0.053 | 0.292 | 0.120 |
| P22735 | TGM1 | 31.645 | MV | 0.340 | 0.103 | 1.403 | 0.173 |
| P24821 | TNC | 0.195 | 0.501 | 2.451 | 0.474 | 0.442 | 0.867 |
| P25311 | AZGP1 | 60.490 | 0.080 | 0.288 | 0.169 | 0.984 | 0.101 |
| P26022 | PTX3 | 0.319 | 1.685 | 0.487 | 1.671 | 0.480 | 0.227 |
| P29508 | SERPINB3 | 25.386 | MV | 0.303 | 0.105 | 2.352 | 0.112 |
| P31944 | CASP14 | 35.769 | 0.131 | 0.450 | 0.179 | 1.507 | 0.047 |
| P35908 | KRT2 | 12.656 | 0.063 | 0.486 | 0.142 | 1.277 | 0.147 |
| P42357 | HAL | 14.316 | MV | 0.024 | 0.121 | 2.658 | 0.084 |
| P47895 | ALDH1A3 | 0.178 | 0.306 | 20.884 | 0.286 | 0.279 | 0.347 |
| Q01469 | FABP5 | 0.281 | 5.778 | 0.325 | 0.443 | 2.158 | 0.695 |
| Q04695 | KRT17 | 16.492 | 0.048 | 0.291 | 0.082 | 1.120 | 0.118 |
| Q08188 | TGM3 | 27.399 | MV | 0.463 | 0.102 | 2.758 | 0.160 |
| Q14112 | NID2 | 0.468 | 5.961 | 0.364 | 4.047 | 0.244 | 0.153 |
| Q15063 | POSTN | 0.097 | 0.497 | 13.710 | 0.704 | 0.358 | 0.875 |
| Q16363 | LAMA4 | 0.460 | 4.004 | 0.247 | 1.348 | 0.377 | 0.173 |
| Q6KB66 | KRT80 | 46.542 | 0.060 | 0.283 | 0.118 | 1.861 | 0.257 |
| Q6UXB8 | PI16 | 0.443 | 0.076 | 2.583 | 0.279 | 1.466 | 0.883 |
| Q8NFJ5 | GPRC5A | 0.294 | 0.490 | 2.664 | 0.831 | 0.418 | 0.481 |
| Q8WVV4 | POF1B | 93.637 | MV | 0.257 | 0.123 | 2.975 | 0.376 |
| Q96RW7 | HMCN1 | 0.366 | 1.505 | 0.292 | 5.357 | 0.383 | MV |
| Q9BT88 | SYT11 | 0.200 | 0.369 | 1.875 | 1.176 | 0.394 | 0.331 |
| Q9BXS4 | TMEM59 | 0.593 | 0.460 | 0.455 | 1.130 | 0.448 | 0.264 |
| Q9NZV1 | CRIM1 | MV | 0.356 | 0.359 | 1.275 | 0.403 | 0.304 |
| Q9UL15 | BAG5 | 0.211 | 0.467 | 1.355 | 0.670 | 0.348 | 0.364 |
| Q9Y666 | SLC12A7 | 0.227 | 0.476 | MV | 0.152 | 0.994 | 0.082 |
